# Activity of *Salvia dolomitica* and *Salvia somalensis* Essential Oils against Bacteria, Molds and Yeasts

**DOI:** 10.3390/molecules23020396

**Published:** 2018-02-13

**Authors:** Valentina Virginia Ebani, Simona Nardoni, Fabrizio Bertelloni, Silvia Giovanelli, Barbara Ruffoni, Carlo D’Ascenzi, Luisa Pistelli, Francesca Mancianti

**Affiliations:** 1 Department of Veterinary Science, University of Pisa, Viale delle Piagge 2, 56124 Pisa, Italy; simona.nardoni@unipi.it (S.N.); fabrizio.bertelloni@vet.unipi.it (F.B.); carlo.dascenzi@unipi.it (C.D.); francesca.mancianti@unipi.it (F.M.); 2 Centro Interdipartimentale di Ricerca “Nutraceutica e Alimentazione per la Salute”, University of Pisa, Via del Borghetto 80, 56124 Pisa, Italy; luisa.pistelli@unipi.it; 3 Department of Pharmacy, University of Pisa, Via Bonanno 6, 56126 Pisa, Italy; silvia.giovanelli84@gmail.com; 4 Centro di Ricerca Orticoltura e Florovivaismo (CREA), Corso Degli Inglesi 508, 18038 Sanremo, Italy; barbara.ruffoni@entecra.it

**Keywords:** *Salvia dolomitica*, *Salvia somalensis*, antibacterial activity, antifungal activity

## Abstract

Essential oils (EOs) from *Salvia dolomitica* and *Salvia somalensis*, widely employed in the cosmetic and perfume industry, were analyzed for composition and tested against bacterial and fungal pathogens isolated from clinical and environmental specimens. The analyses were carried out against *Staphylococcus aureus*, *Staphylococcus pseudointermedius*, *Pseudomonas aeruginosa*, *Escherichia coli*, *Streptococcus canis*, *Streptococcus pyogenes*, *Klebsiella pneumoniae*, *Proteus mirabilis*, *Microsporum canis*, *Microsporum gypseum*, *Trichophyton mentagrophytes*, *Aspergillus niger*, *Aspergillus flavus*, *Candida albicans*, *Candida krusei*, *Mucor* sp. and *Trichothecium roseum*. Both EOs showed similar percentages of total monoterpenes and sesquiterpene hydrocarbons. The main constituents were 1,8-cineole and *β*-caryophyllene in *S*. *dolomitica* and bornyl acetate and camphor in *S*. *somalensis*. The selected EOs have no relevant antifungal or antibacterial activities if compared to conventional drugs.

## 1. Introduction

The genus *Salvia* belongs to the family Lamiaceae and includes about 900 species spread throughout the world. Taxa of *Salvia* spp. (sage) are mainly aromatic plants, rich in essential oils (EOs) and widely employed in foods, perfumery and cosmetics [1]. EOs from *Salvia* spp. are rich in monoterpene hydrocarbons (MH), oxygenated monoterpenes (OM) and sesquiterpenes [2].

Natural products isolated from *Salvia* spp. have long been used in traditional medicine to treat several microbial afflictions [2], but considering the high number of species of this genus, the antimicrobial properties of all of them is not well known. *Salvia dolomitica* Codd and *Salvia somalensis* Vatke are two species native to Africa. The first sage is an aromatic perennial shrub able to grow on heavy soils with dolomitic rocks. EO from *S*. *dolomitica* has been suggested as an antiplasmodial and antiinflammatory remedy. Its antimicrobial activity against some bacteria has been described [3]. *S*. *somalensis* is a wild sage native to Somalia. A potential cosmetic application of its EO has been proposed for its peculiar olfactive characters [4], but data about its antimicrobial activity are not available in the scientific literature. Considering that EOs from these botanical species are already employed, the aim of the present investigation was to verify the activity of EOs from *S*. *dolomitica* and *S*. *somalensis* against bacterial and fungal pathogens previously isolated from clinical and environmental specimens. These organisms were selected for their relevance in veterinary practice and/or in foodstuff preservation.

## 2. Results

### 2.1. EO Analysis

The EOs’ composition is reported in Table 1. A total of 59 compounds were characterized, with identification percentages ranging from 98.5% in *S*. *somalensis* to 98.9% in *S*. *dolomitica*. The monoterpenes class was the most abundant in both samples (73.1% in S. *dolomitica* and 67.8% in *S*. *somalensis*). 1,8-Cineole was the main constituent in *S*. *dolomitica* (18.9%), while bornyl acetate (16.1%) together with camphor (12.5%), showed the highest percentage in *S*. *somalensis*. Among the sesquiterpenes, sesquiterpene hydrocarbons (SH) exhibited a higher relative amount in comparison with the oxygenated sesquiterpenes (OS) in both samples. *β*-Caryophyllene predominated in *S*. *dolomitica* (13.1%) and *δ*-cadinene in the other sage sample (6.4%). 

### 2.2. Antibacterial Activity

#### 2.2.1. Agar Disk Diffusion Method

The agar disk diffusion method showed that EO from *S*. *somalensis* did not inhibit any tested bacterial isolate; conversely *S*. *dolomitica* EO had a low effectiveness at 10% dilution (MIC 16.26 mg/mL) against *Streptococcus canis*, *Klebsiella pneumoniae* and *Proteus mirabilis*. No inhibition zone was observed for the negative control. Results of the Kirby-Bauer assay are reported in Table 2.

#### 2.2.2. Minimum Inhibitory Concentration

Similar results were obtained by the broth microdilution method. In fact, MIC values of 16.26 mg/mL were observed when *S*. *dolomitica* EO was tested against *S*. *canis*, *K*. *pneumoniae* and *P*. *mirabilis*, whereas bacterial growth was found for the remaining isolates. All bacterial strains were not inhibited by *S*. *somalensis* EO. Bacterial growth was observed when bacteria had been incubated with brain hearth infusion broth (BHIB, Oxoid Ltd., Basingstoke, Hampshire, UK) without EOs, whereas it was not detected in the sterility control wells.

### 2.3. Antifungal Activity

The examined EOs showed variable degrees of antimycotic activity towards tested fungal isolates. From a general point of view, molds appeared more prone to the selected EOs rather than yeasts. *Microsporum gypseum* appeared to be the most sensitive fungal species, inhibited by 1.70 mg/mL of *S*. *somalensis* EO with IC_50_ 0.84. Moreover, *Mucor* sp. and *Trichothecium roseum* did not show a visible growth at 4.06 mg/mL (IC_50_ 2.07) of *S*. *dolomitica*, and 5.11 mg/mL (IC_50_ 4.42) of *S*. *somalensis*, respectively. *Candida* spp. and *Aspergillus* spp. employed were susceptible to selected conventional drugs. Mycotic growth was observed in negative control wells (without EOs). All the results of EOs and results of conventional drugs, with available clinical breakpoints are reported in Table 3.

## 3. Discussion

*S*. *dolomitica* Codd is an aromatic perennial shrub, native of the North-East province of Transvaal in Southern Africa. The species name refers to its frequent occurrence on soil with dolomite rock. This sage is highly scented and drought resistant [5]. *S*. *dolomitica* is mainly commercialized for ornamental purposes and can be cultivated in pots and used as groundcover, shrub border or edging [6].

Fisher [7] and Kamatou et al. [8] characterized EOs from fresh aerial parts of *S*. *dolomitica* Codd grown in open fields in South Africa. The comparison between the analysis of the EO obtained from *S*. *dolomitica* grown in the Cape region of South Africa and our plant collected in Sanremo (Italy) showed a different behaviour, since the South African sample had a high percentage of geraniol (19.6%) and linalyl acetate (19.6%), totally absent in our sample, followed by linalool and *α*-terpineol (16.6% and 6.2%, respectively) [8]. The composition of our *S*. *dolomitica* EO also differed from the other South African sample [7] for the amounts of main constituents. In detail South African sample was composed of 17.6% of *β*-caryophyllene, 17.4% of 1,8-cineole, 9.7% of limonene, 8.5% of borneol, 7.5% of *δ*-3 carene and 7.1% of *α*-pinene. *S*. *dolomitica* EO of the present study showed almost the same compounds in different amounts: 18.9% of 1,8-cineole, 13.2% of *β*-caryophyllene, 9.6% of *α*-pinene, 8.9% of limonene, 6.7% of *δ*-2 carene, 6.6% of borneol and 5.2% camphene.

*S*. *somalensis* Vatke EO, recovered after hydrodistillation of the dried aerial parts, has its main practical application in the cosmetic industry. This taxon is a wild plant native to Somalia, where it grows in full sun to partial shade. *S*. *somalensis* is characterized by a pleasant fragrance and it is well known as easy-to-grow ornamental plant especially in dry gardens, due to its drought tolerance [4]. The EO obtained from *S*. *somalensis* showed a chemical profile very similar to that reported by Villa et al. [4]. However *δ*-cadinene and *α*-copaene had higher percentages in our sample (6.43% versus 0.26% and 3.46% versus 1.43%, respectively). 

To the best of our knowledge no studies about the antimicrobial activity of *S*. *somalensis* are reported in the scientific literature. In the present paper, *S*. *somalensis* was ineffective against all the tested bacterial strains, while *S*. *dolomitica* had a very low activity against *S*. *canis*, *K*. *pneumoniae* and *P*. *mirabilis*. 

The EOs examined in the present study are characterized by different composition, apart from their content in *α*-pinene. Bornyl acetate and camphor were the main constituents in *S*. *somalensis*, compounds totally absent in *S*. *dolomitica* EO, where 1,8-cineole and *β*-caryophyllene showed the highest percentages. These pure compounds are known to be active as antimicrobial agents [9,10]. 

Little information is available about the antimicrobial activity of *S*. *dolomitica*. Fisher [7] reported a slight activity of *S*. *dolomitica* EO against *Bacillus cereus*, but not against *Escherichia coli*, *S*. *aureus*, *K*. *pneumoniae* and *Yersinia enterocolitica*.

Previous investigations on other *Salvia* spp. reported antimicrobial activity of *Salvia sclarea* EO against *P*. *aeruginosa* and mainly *S*. *pseudointermedius* [11], whereas *S*. *dolomitica* and *S*. *somalensis* EOs tested herein had no inhibition activity against these bacterial strains.

On the basis of the results obtained in previous studies, Gram positive bacteria would seem to be more sensitive to *Salvia* spp. EOs than Gram negative bacteria [12,13]. The present investigation does not confirm this hypothesis, because inhibition activity of *S*. *dolomitica* was observed against both Gram positive (*S*. *canis*) and Gram negative (*K*. *pneumoniae* and *P*. *mirabilis*) bacteria.

All the tested bacteria used in this work were clinical isolates. In fact, these species can be frequently found in cases of septicemia and infections of ears, skin, genitourinary and gastrointestinal tracts. Treatment with antibiotics is usually performed, even though the infection’s resolution is not obtained in some cases because of the antibiotic-resistance of the etiologic agents.

To the best of our knowledge this is the first study dealing with the antimycotic activity of these *Salvia* species. The antifungal action of the EOs yielded some interesting findings. *S*. *dolomitica* and *S*. *somalensis* EOs appeared active against *Mucor* sp. and *T*. *roseum*, respectively. These fungi are spoiling molds of foodstuff, so a possible use of the investigated EOs as food additives could be suggested. Small amounts of both EOs appeared to be active against *M*. *gypseum*, a dermatophyte responsible for ringworm both in animals and in humans. Furthermore, this mold is reported to possess a limited sensitivity versus conventional antimycotic drugs such as griseofulvin [14] and itraconazole [15]. For this reason these compounds could be evaluated as active ingredients of shampooing/lotion formulations intended for local application. The major components of both examined EOs such as 1,8 cineole, bornyl acetate, *β*-caryophyllene, *α*-pinene, camphor were shown to possess antifungal activity [16], in particular EOs containing such components were effective against *Fusarium* spp. [17], *Heterobasidion parviporum* [18] and EOs from plants rich in bornyl acetate and *α*-pinene showed antimycotic action [19,20].

*β*-Caryophyllene is reported to have a fungicide activity versus *Aspergillus niger* and *Aspergillus fumigatus* [21], while 1,8 cineole has showed high MICs against *Microsporum canis* and *M*. *gypseum* [15]. The EOs used in the present study did not give good results when assayed against aspergilli, in contrast with a previous report referred to *S*. *sclarea*, while the lack of activity against *Candida* yeasts was confirmed [11]. However, data from the literature showed that *S*. *officinalis* was active against yeasts, aspergilli and dermatophytes [22], while *Salvia fruticosa* was highly effective versus dermatophytes [23] and phytopathogenic fungi [24].

Some papers are referred to EOs from plants in which such active principles were present in higher amounts [17]. Being represented in amounts lower than 20%, they could be responsible for their moderate antifungal effect.

Sage EOs have been proposed for antimicrobial treatments [3]. However, several species of the genus *Salvia* occur worldwide and their different chemical compositions have been demonstrated in several studies [25]. These differences could be due to several factors, including their habitat, cultivation methods and extraction procedures. Thus the antimicrobial activity of different botanical species should be thoroughly verified, according to their chemotype.

## 4. Material and Methods

### 4.1. Essential Oils

*S*. *dolomitica* Codd (Voucher number PI010354) and *S*. *somalensis* Vatke (Voucher number PI010355) belong to a collection coming from the CREA (Centro di ricerca Orticoltura e Florovivaismo) located in Sanremo (Liguria, Italy) and collected in 2015. Both the *Salvia* spp. were vegetatively propagated, planted and grown under uniform conditions. The aerial parts were dried and hydrodistillated for 2 h to obtain the respective EOs, that were maintained at 4 °C in dark glass vials and microbiologically tested for quality control before use.

### 4.2. Gas Chromatography-Mass Spectrometry Analysis

The GC-MS analysis and the identification of the constituents in both EOs were performed following a previously reported protocol [26].

### 4.3. Antibacterial Activity

#### 4.3.1. Bacterial Strains

Both EOs were tested against the following bacterial species: *S*. *aureus*, *S*. *pseudointermedius*, *P*. *aeruginosa*, *E*. *coli*, *S*. *canis*, *S*. *pyogenes*, *K*. *pneumoniae*, *P*. *mirabilis*. All the strains have been previously isolated from clinical specimens collected from dogs with different pathologies, such as infection of ear, skin and genitourinary tract.

#### 4.3.2. Agar Disk Diffusion Method

The antibacterial activity of both EOs was determined with the Agar disk diffusion method following the procedures described by Clinical and Laboratory Standards Institute (CLSI) [27] and with some modifications as previously described [28]. Each EO was tested at a 1:10 dilution in dimethyl sulfoxide (DMSO, Oxoid Ltd.). An absorbent paper disc impregnated with 10 µL of DMSO was used as negative control. Paper discs impregnated with amikacin (30 µg), amoxycillin-clavulanic acid (30 μg), cefotaxime (30 µg), tetracycline (30 µg) (Oxoid Ltd.), respectively, were used as positive controls. The results of the bacterial in vitro sensitivity to antibiotics were interpreted as indicated by the CLSI [29]. All tests were performed in triplicate.

#### 4.3.3. Minimum Inhibitory Concentration 

Each bacterial strain was also submitted to MIC determination. MIC was evaluated by the broth microdilution method following the protocol previously reported [28]. The test was performed in a total volume of 200 µL with final EOs concentrations of 16.26 to 2.03 mg/mL for *S*. *dolomitica*, 17.04 to 2.13 mg/mL for *S*. *somalensis*. The same assay, carried out in BHIB was simultaneously performed for bacterial growth control (tested bacteria and BHIB) and sterility control (tested oil and BHIB). All tests were performed in triplicate.

### 4.4. Antifungal Activity

#### 4.4.1. Fungal Strains

Isolates of dermatophytes (*M*. *canis*, *M*. *gypseum*, *Trichophyton mentagrophytes*) and yeasts (*Candida albicans* and *Candida krusei*) were obtained from animal specimens. Other molds such as *A*. *niger*, *Aspergillus flavus*, *Mucor* sp. and *T*. *roseum* were isolated from environmental sources and were used for in vitro sensitivity assays. These fungi were selected for their impact on both animal and human health. In particular dermatophytes are keratinophilic and keratinolytic molds able to infect both animal and human teguments and annexes, responsible for ringworm. *C*. *albicans* and *C*. *krusei* are endo- and esosaprophytic yeasts, respectively, known as causative agents of superficial mycoses (stomatitis, vulvovaginitis) in immunocompetent hosts, and deep generalized life threatening infections in immunocompromised patients. *A*. *niger*, *A*. *flavus*, *Mucor* sp. and *T*. *roseum* are environmental food-spoiling molds. Furthermore *A*. *flavus*, and *T*. *roseum* are toxigenic fungi, able to elaborate secondary metabolites, such as aflatoxins and trichothecenes on foodstuff, which can cause severe chronic lesions in consumers. All molds (dermatophytes and environmental fungi) were maintained on Potato Dextrose Agar at −20 °C, while yeasts were stored into sterile distilled water.

#### 4.4.2. Microdilution Test

The antimycotic activity of EOs was essayed by a microdilution test carried out as recommended by Clinical and Laboratory Standards Institute M38A_2_ [30] for molds and Clinical and Laboratory Standards Institute M27A_3_ [31] for yeasts, starting from a 10% dilution corresponding to 17.04 mg/mL (*S*. *somalensis*) and 16.26 mg/mL (*S*. *dolomitica*), respectively, and 7.5%, 5%, 3%, 2% and 1% further dilutions were achieved. All assays were performed in triplicate. Controls were also achieved using conventional antifungal drugs. Clinical breakpoint of standard drugs referred to *Aspergillus* and *Candida* species provided by CLSI were taken into consideration.

## 5. Conclusions

Our results show that *S*. *somalensis* and *S*. *dolomitica* EOs, largely employed in cosmetic and perfume industry, have not relevant both antifungal and antibacterial activities if compared to conventional drugs. So these EOs cannot be considered promising candidates not even against fungal organisms with lower sensitivity such as *M*. *gypseum*, *Mucor* sp. and *T*. *roseum*.

## Figures and Tables

**Table 1:** EO analysis of *Salvia dolomitica* and *Salvia somalensis* aerial parts.

No.	Class	Component	L.R.I. ^§^	Relative Content (%)	
				*S*.* dolomitica*	*S*.* somalensis*
1	MH	*α*-Thujene	930	0.25	tr
2	MH	Tricyclene	932	0.10	tr
3	MH	*α*-Pinene	939	9.60	9.28
4	MH	Camphene	955	5.23	8.04
5	MH	Sabinene	978		0.77
6	MH	*β*-Pinene	981	1.31	
7	MH	Myrcene	993	2.73	1.23
8	MH	*δ*-2-Carene	1002	6.68	6.29
9	MH	*α*-Phellandrene	1006	0.82	0.47
10	MH	*α*-Terpinene	1019	0.95	0.48
11	MH	*p*-Cymene	1025	1.55	1.55
12	MH	Limonene	1032	8.94	4.62
13	OM	1,8-Cineole	1036	18.91	tr
14	MH	( *Z*)-*β*-Ocimene	1037	2.66	tr
15	MH	( *E*)-*β*-Ocimene	1053	0.39	tr
16	MH	*γ*-Terpinene	1058	1.13	0.47
17	MH	*para*-Mentha-2,4(8)-diene	1088	0.39	0.14
18	MH	Terpinolene	1090	0.21	0.74
19	OM	Linalool	1102	0.17	0.23
20	OM	*trans*-Thujone	1117	0.10	
21	OM	Camphor	1148		12.52
22	OM	Borneol	1169	6.57	2.79
23	OM	4-Terpineol	1180	0.59	0.67
24	OM	*α*-Terpineol	1192	0.11	1.26
25	NT	( *Z*)-Butanoic acid	1236	0.12	
26	OM	Bornyl acetate	1289		16.15
27	SH	*α*-Cubebene	1351	0.20	0.31
28	SH	Cyclosativene	1371		0.68
29	SH	Isoledene	1376	0.31	
30	SH	*α*-Copaene	1377	1.69	3.46
31	OM	( *Z*)-Jasmone	1397		0.10
32	SH	Cyperene	1399	0.55	
33	SH	*α*-Gurjunene	1410		0.89
34	SH	*β*-Caryophyllene	1418	13.12	4.52
35	SH	*β*-Gurjunene	1434	0.62	0.19
36	SH	*α*-Guaiene	1440	0.46	1.37
37	SH	*cis*-Muurola-3,5-diene	1450		0.11
38	OM	*trans*-Carvyl propanoate	1455	3.68	
39	SH	*α*-Humulene	1456	1.08	0.35
40	SH	*allo*-Aromadendrne	1460	0.31	0.43
41	SH	*trans*-Cadina-1(6),4-diene	1477	0.35	0.59
42	SH	*γ*-Muurolene	1480	0.37	0.78
43	SH	*γ*-Curcumene	1481		0.24
44	SH	Germacrene D	1484		0.5
45	SH	*β*-Selinene	1490	0.21	0.1
46	SH	*cis*-*β*-Guaiene	1493	0.13	0.17
47	SH	Valencene	1496	1.43	0.73
48	SH	Viridiflorene	1497	0.51	
49	SH	*trans*-*β*-Guaiene	1503		0.88
50	SH	*trans*-*γ*-Cadinene	1513	1.35	3.11
51	SH	*δ*-Cadinene	1523	2.54	6.43
52	SH	*trans*-Cadina-1(2),4-diene	1535	0.24	0.56
53	SH	*α*-Cadinene	1539	0.10	0.25
54	SH	*α*-Calacorene	1546	tr	0.37
55	OS	*trans*-Nerolidol	1566		0.64
56	OS	Caryophyllene oxide	1583		0.14
57	OS	1- *epi*-Cubenol	1630	tr	0.66
58	OS	Hinesol	1638	0.14	1.99
59	OS	Cubenol	1641		0.21
Non terpenic compounds (NT)				0.12	0.00
Monoterpene hydrocarbons (MH)				42.94	34.08
Oxygenated monoterpenes (OM)				30.13	33.72
Total monoterpenes (TM)				73.07	67.80
Sesquiterpene hydrocarbons (SH)				25.57	27.02
Oxygenated sesquiterpenes (OS)				0.14	3.64
Total sesquiterpenes (TS)				25.71	30.66
Total identified				98.90	98.46

^§^: Linear retention index.

**Table 2:** Results (inhibition zone expressed in mm) of the Kirby-Bauer assay testing the selected bacterial strains against four antibiotics.

		Antibiotics (30 µg)		
Bacterial Strains	Amikacin	Amoxycillin + Clavulanic Acid	Cefotaxime	Tetracycline
*Staphylococcus aureus*	18 (S)	20 (S)	16 (I)	12 (R)
*Staphylococcus pseudointermedius*	19 (S)	21 (S)	23 (S)	11 (R)
*Streptococcus canis*	19 (S)	21 (S)	23 (S)	20 (S)
*Streptococcus pyogenes*	20 (S)	23 (S)	23 (S)	20 (S)
*Pseudomonas aeruginosa*	12 (R)	20 (S)	17 (I)	12 (R)
*Escherichia coli*	13 (R)	22 (S)	25 (S)	21 (S)
*Proteus mirabilis*	20 (S)	22 (S)	24 (S)	20 (S)
*Klebsiella pneumoniae*	15 (I)	20 (S)	24 (S)	10 (R)

Legend—S: susceptible; R: resistant; I: intermediate. Breakpoint values: amikacin ≤ 14 mm R, 15–16 mm I, ≥17 mm S; amoxicillin + clavulanic acid ≤ 19 mm R (staphylococci), ≤13 mm R (remaining bacteria), 14–17 mm I, ≥20 mm (staphylococci) S, ≥18 mm (remaining bacteria) S; cefotaxime ≤ 14 mm R, 15–22 mm I, ≥23 mm S; tetracycline ≤ 14 mm R, 15–18 mm I, ≥19 mm S.

**Table 3:** Results of microdilution assay testing *S*. *somalensis* and *S*. *dolomitica* EOs and conventional antimycotic drugs against fungal strains from different sources.

		Essential Oils					
Fungal Strains	Source of Isolates	*S*. *somalensis*		*S*. *dolomitica*		Conventional Drugs (mg/mL)	CLSI Clinical Breakpoint
		MIC (mg/mL)	IC_50_ (mg/mL)	MIC (mg/mL)	IC_50_ (mg/mL)		
*Microsporum canis*	feline hair	17.04	12.8	16.26	11.62	ITZ (0.125)	-
*Microsporum gypseum*	feline hair	1.70	0.84	4.88	4.26	ITZ (32)	-
*Trichophyton mentagrophytes*	canine hair	17.04	12.8	16.26	11.62	ITZ (32)	-
*Aspergillus niger*	environment	8.52	7.84	8.13	4.48	AmB (1.5)	2
*Aspergillus flavus*	environment	17.04	12.8	16.26	11.62	AmB (1)	2
*Candida albicans*	canine feces	17.04	12.8	16.26	11.62	FLU (0.25 × 10^−3^)	8
*Candida krusei*	canine feces	17.04	12.8	16.26	11.62	AmB (0.5)	1
*Mucor sp*.	environment	8.52	7.84	4.06	2.07	AmB (0.5)	-
*Trichothecium roseum*	environment	5.11	4.42	8.13	4.48	Thiabendazole (4)	-

Legend—ITZ: itraconazole; FLU: Fluconazole; AmB: Amphotericine B; CSP: Caspofungin.
